# Diagnostic and Therapeutic Options in Myocarditis and Inflammatory Cardiomyopathy

**DOI:** 10.3390/biomedicines14030691

**Published:** 2026-03-17

**Authors:** Heinz-Peter Schultheiss, Felicitas Escher, Ganna Aleshcheva, Gordon Wiegleb, Christian Baumeier

**Affiliations:** 1 Institute of Cardiac Diagnostics and Therapy, IKDT GmbH, 12203 Berlin, Germany; 2 Deutsches Herzzentrum der Charité, Department of Cardiology, Angiology and Intensive Care Medicine Campus Virchow-Klinikum, Augustenbruger Platz 1, 13353 Berlin, Germany; 3 Charité—Universitaetsmedizin Berlin, corporate member of Freie Universitaet Berlin and Humboldt-Universitaet zu Berlin, Charitéplatz 1, 10117 Berlin, Germany; 4 DZHK (German Centre for Cardiovascular Research), Partner Site, 10785 Berlin, Germany

**Keywords:** myocarditis, inflammatory cardiomyopathy, endomyocardial biopsy, advanced diagnostics, personalized treatment

## Abstract

Myocarditis and inflammatory cardiomyopathy are inflammatory diseases of the heart muscle that can have both infectious and non-infectious causes. They can be caused by an unresolved viral infection or other infection, or they can be autoimmune, toxic, or allergic in nature. The specific identification of the pathogen and/or confirmation of inflammation can only be achieved through direct tissue analysis using endomyocardial biopsy (EMB), as neither detection of the virus nor assessment of the quality and intensity of the inflammation is possible using non-invasive methods. Accordingly, the removal and analysis of an EMB is considered the diagnostic gold standard in international guidelines and statements. The sudden onset of atypical angina pectoris and initially exertion-dependent dyspnea, as well as arrhythmias, pericardial effusion, and progressive symptoms of heart failure, indicate an acute inflammatory process of the myocardium. In addition, nonspecific symptoms such as fatigue and reduced physical performance may also occur. Diagnostic evaluation includes an electrocardiogram (ECG), cardiac imaging, and laboratory tests. The analysis of the EMB is crucial for a definitive diagnosis and thus for the initiation of an etiology-based, specific and personalized therapy. This includes histological and immunohistochemical inflammation diagnostics as well as molecular virological diagnostics. These enable both the detection of viruses and the assessment of transcriptional virus activity. New analyses using metagenomic next generation sequencing (NGS) techniques provide insights of enormous diagnostic and therapeutic relevance. This applies both to the spectrum of detectable pathogens and to the possibility of confirming transcriptional viral activity. In addition, gene expression profiling enables the differentiation of specific forms of myocardial inflammation (e.g., giant cell myocarditis, cardiac sarcoidosis, and eosinophilic myocarditis) and reduces the influence of “sampling errors” in focal inflammatory processes. The treatment of heart failure or ventricular arrhythmias is always symptomatic according to general evidence-based guidelines. In severe cases, mechanical circulatory support or even a heart transplant may be necessary. Patients with histologically confirmed myocardial inflammation or intramyocardial viral infection can be offered specific, causal, and personalized therapy. These patients can be successfully treated with immunosuppressive or antiviral therapy, which significantly improves the prognosis of the disease.

## 1. Introduction

Myocarditis is an inflammatory disease of the heart muscle with a wide range of infectious and non-infectious causes, and it can occur at any age. Although, in principle, any infectious pathogen can affect cells of the myocardium (such as cardiomyocytes, fibroblasts, or endothelial cells), viruses are most commonly identified as the causative agents of acute myocarditis and inflammatory cardiomyopathies in industrialized countries [1,2,3,4].

Chronic inflammation, which manifests as inflammatory (dilated) cardiomyopathy, is defined by impaired systolic function and ventricular dilation and develops when acute viral inflammatory processes do not resolve spontaneously. In addition, post-infectious or autoimmune inflammatory processes (mediated by autoantibodies or immune cells), often occurring in the context of systemic diseases, also play a significant role.

A definitive diagnosis based on the analysis of myocardial biopsies is essential for clarifying the differential diagnosis and implementing specific treatment strategies. Non-invasive diagnostic methods are not capable of classifying and quantifying the inflammatory process or detecting viruses with or without transcriptional viral activity [5,6].

Myocardial inflammation due to bacterial, protozoal, or fungal infections is less common and more frequently observed in developing countries. Other, albeit rare, causes include rheumatic or granulomatous systemic diseases (e.g., tuberculosis, giant cell myocarditis, or sarcoidosis), myocarditis triggered by allergic reactions to medications, as well as damage caused by heavy metals, toxins, and physical noxae (Table 1) [7].

The pathophysiological processes in both infectious and non-infectious myocarditis occur at the cellular and subcellular level. The specific identification of pathogens and/or inflammation can only be achieved through direct analysis of myocardial tissue, which serves as the basis for a causal, specific, and personalized therapy. The required diagnostic material can be readily obtained by means of an endomyocardial biopsy (EMB).

**Figure 1:**
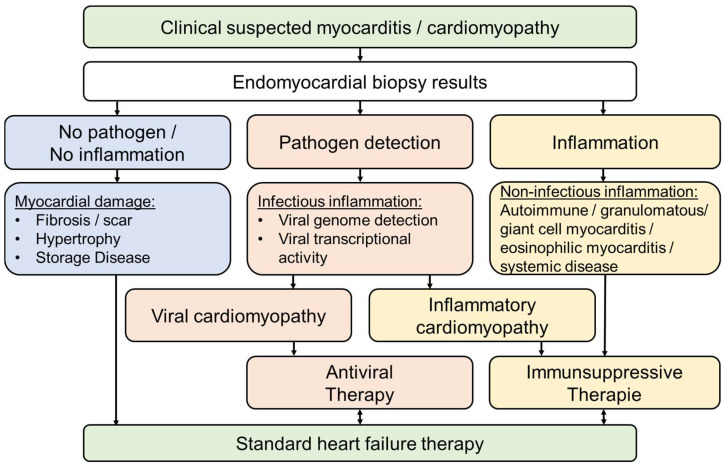
Schematic representation of the diagnostic workflow using endomyocardial biopsy (EMB) in cases of suspected myocarditis or cardiomyopathy. In addition to histopathological examinations for fibrosis, hypertrophy, and storage disorders (blue), pathogens (red) and inflammatory activity (yellow) are analyzed. In cases of infectious inflammation, antiviral therapy will be initiated. In contrast, virus-negative EMB with evidence of inflammation indicates the use of immunosuppressive therapy. If neither pathogen nor inflammation are detected, patients can be treated with standard heart failure therapy.

For differential therapeutic considerations, it is crucial that a positive treatment effect can only be achieved if treatable causes—such as viral infections, inflammatory processes, or cardiodepressive autoantibodies—are precisely identified and characterized (Figure 1). Moreover, the timing of the diagnostic evaluation is of critical importance to ensure that the myocardium still possesses regenerative potential [8]. If diagnostics are only performed after irreversible myocardial damage has already occurred—as is the case in infectious or inflammatory dilated cardiomyopathy—the development or progression of chronic heart failure can no longer be prevented in the long-term, regardless of the specific underlying disease process. This highlights the necessity of performing specific diagnostics, such as EMB, as early as possible in order to ensure causal and targeted diagnosis [9]. Moreover, genetic predisposition plays a significant role, with Toll-like receptors and genetic variants appearing to be important factors in the development of myocarditis [10,11].

## 2. Pathophysiology

When an infectious pathogen invades an organism, the immune system is activated to inactivate the potentially harmful microbe systemically before organs such as the heart can be affected and damaged. If this fails, inflammatory cells migrate into the myocardium via blood vessel endothelia activated in the infected tissue areas (expressing cell adhesion molecules) and guided by cytokine gradients, triggering an initially localized inflammatory response (focal myocarditis) in the infected myocardial regions [12]. In this process, infiltrating effector cells (lymphocytes, macrophages) recognize and destroy virus infected cardiomyocytes (active myocarditis) in order to rapidly eliminate the pathogens and prevent further spread. The myocardial damage detectable in this early phase is therefore caused not only by cytolytic viral infections themselves but also by activated immune defense mechanisms of the innate and adaptive immune systems. If only a few cardiomyocytes are affected, the remaining organ damage after resolution of the inflammation is usually minimal. Global myocardial function is either not impaired or only mildly reduced. However, since this often results in defective healing, even small scars can serve as a starting point for persistent arrhythmias.

If larger defects (fibrosis, scar areas) caused by viral or inflammatory processes have developed, myocardial function remains impaired even if the inflammation has spontaneously resolved following successful virus elimination or if immunosuppressive therapy has been effective in virus-negative inflammation (post-infectious or post-inflammatory cardiomyopathy). The extent of the remaining pump dysfunction and, in the long-term, the prognosis therefore depend on the degree of irreversible myocardial damage caused by the viral infection and inflammatory response. This underscores the importance of an early, etiopathogenetic diagnosis in order to establish the basis for causal and specific therapy (Figure 1).

Heart failure progressing over weeks, months, or years can develop on the basis of a chronic infection or inflammation, but also if the immune system fails to eliminate the infectious agent from the heart (chronic viral cardiomyopathy), or if the inflammatory process does not spontaneously resolve after successful virus elimination (chronic inflammatory cardiomyopathy).

The variable symptomatology of viral myocarditis and its course are based on the different cardiac infection patterns of the various infectious agents [13]. When cardiomyocytes are primarily affected (enterovirus/adenovirus infections), this sooner or later leads to systolic pump dysfunction [14]. The life expectancy of these patients is significantly reduced due to heart failure and/or arrhythmias, even when all available symptomatic treatment options are optimally utilized [15,16].

Erythroparvoviruses and various herpesviruses are predominantly found in endothelial cells, which regulate the blood supply to the heart muscle in the microcirculation [17]. The symptoms of these patients are based on virus-associated endothelial damage (endothelial dysfunction) or the loss of capillaries. As a result, especially during exertion, the increased oxygen demand cannot be met due to vascular spasms or vascular rarefaction [18,19]. Angina pectoris, shortness of breath, and reduced exercise capacity are the consequences. Since the myocytes responsible for the heart’s pumping function are not directly affected, cardiac performance often remains preserved for a long time. In the long-term, persistent microangiopathy leads to chronic myocardial ischemia, which subsequently causes increasing fibrosis and thereby results in impaired pumping function [20].

## 3. Epidemiology

Estimates suggest an incidence of myocarditis of 1–10 cases per 100,000 inhabitants per year. However, due to the lack of systematic studies and especially biopsy-based data (the diagnostic gold standard), no precise data are available. This is also due to the fact that the often uncharacteristic and nonspecific symptoms cause the majority of subacute myocarditis and inflammatory cardiomyopathies to go initially unrecognized and undiagnosed.

Myocarditis can occur in all age groups but is most commonly observed between 20 and 60 years of age. Men of all age groups are significantly more affected than women. Autopsy studies of children and adolescents with sudden cardiac death showed a highly variable proportion of myocarditis ranging from 2 to 42% [21,22]. Biopsy-based studies revealed a myocardial inflammatory process in 46% of children [23] and in 9–16% of adults with dilated cardiomyopathy (DCM) [24,25]. Inflammatory cardiac involvement due to non-infectious autoimmune processes occurs in approximately 10% of cases in systemic diseases [26].

In Lyme disease, cardiac involvement is seen in approximately 8% of cases. Clinically more significant are virus-associated inflammations as well as post-infectious auto-inflammatory myocardial processes, where the inflammation leading to virus elimination does not resolve normally. Various viral pathogens are detected molecularly, with or without accompanying inflammation, in about 50% of the patients examined [15,23,27].

## 4. Clinics

### 4.1. Acute Myocarditis

Key symptoms of acute myocarditis: Sudden onset of atypical angina pectoris and initially mainly exertion-induced dyspnea, along with newly documented arrhythmias (clinically palpitations or tachycardia), pericardial effusion, and progressive heart failure symptoms indicate an acute inflammatory process, especially when these symptoms appear for the first time after an infection in previously heart-healthy patients and no other diseases are present. Severe chest pain when lying down that improves upon sitting up is indicative of pericardial involvement (perimyocarditis, pericarditis).

Uncharacteristic symptoms: In addition to the above more typical symptoms of cardiac inflammation, uncharacteristic complaints such as fatigue, tiredness, and reduced physical capacity may also occur. These are often misinterpreted as consequences of a prior pulmonary or gastrointestinal infection, which about 80% of patients experienced 2–4 weeks earlier. If these are the only symptoms, potential cardiac involvement is often overlooked by both patients and doctors, and specific diagnostics are started too late or, more frequently, not at all [28].

Special diagnostic constellations: Less frequently, acute myocarditis manifests with a rapidly progressive impairment of pump function. If ventricular arrhythmias such as tachycardias or ventricular fibrillation occur simultaneously, the rare form of giant cell myocarditis should be considered. Giant cell myocarditis is a rare autoimmune myocarditis of unclear etiology that is often underdiagnosed due to focal infiltration and has a very poor prognosis if left untreated. Rapid diagnostic confirmation via EMB, including analysis of gene expression profiles to circumvent the commonly observed “sampling error,” is essential to avoid delaying the life-saving immunosuppressive therapy.

Rapid deterioration of pump function, which—unlike giant cell myocarditis—is less frequently accompanied by life-threatening arrhythmias, also occurs in eosinophilic myocarditis and granulomatous heart diseases such as cardiac sarcoidosis. Blood eosinophilia may not be present in the early stage of cardiac decompensation, so the diagnosis is usually established through biopsy diagnostics. Granulomas often exhibit a focal distribution pattern. Positron emission tomography (PET) scan and gene expression profile analysis can provide important additional information regarding cardiac involvement, since histological detection of granulomas in myocardial biopsies often fails due to the focal nature of the disease [29,30,31,32,33,34,35,36].

### 4.2. Subacute/Chronic Myocarditis and/or Inflammatory Cardiomyopathy

Rarely is the clinical presentation so pronouncing that patients consult their physician early enough for characteristic findings of acute myocardial injury to be present. In this subacute stage of the disease, patients are more often noted due to nonspecific ST-segment changes or T-wave inversions, conduction or impulse formation disturbances, or supraventricular or ventricular extrasystoles, or they report persistent sinus tachycardia or slow normalization of the pulse after exertion.

## 5. Diagnostics

### 5.1. Electrocardiography

The electrocardiogram (ECG), though only 47% sensitive, is often the first screening tool for myocarditis [37]. Normal or nonspecific ECGs do not rule it out, but pathological findings may include Q waves, left bundle branch block, wide QRS (>120 ms), prolonged QT, AV block, malignant tachyarrhythmias, and T-wave inversions [38,39,40].

### 5.2. Laboratory

Biomarkers assist in diagnosis but lack specificity. Acute myocarditis can mimic infarction with angina-like symptoms, ST elevations, and elevated troponin. Viral IgM indicates recent infection but does not confirm myocardial involvement, and polyclonal serological responses are common [41,42]. Viral serology alone is not diagnostic or treatment-guiding [43].

### 5.3. Echocardiography

Transthoracic echocardiography is widely available and screens for LV dysfunction but cannot confirm myocarditis. Findings may include LV dilation, reduced function, wall thickening, regional motion abnormalities, diastolic dysfunction, or pericardial involvement [44,45]. Speckle-tracking strain analysis adds diagnostic and prognostic value, but echocardiography cannot determine etiology [46].

### 5.4. Cardiac Magnetic Resonance Imaging

Cardiac magnetic resonance (CMR)-based tissue characterization can provide indications of inflammatory myocardial disease (Lake Louise criteria) [47]. It has high diagnostic value in detecting myocarditis, especially in the acute phase during the first few weeks. As a consequence, CMR has evolved in the 2025 ESC guidelines from a primarily supportive imaging modality to a recommended Class I non-invasive diagnostic tool that can be used to make a definitive clinical diagnosis of myocarditis [48]. However, its sensitivity and specificity relative to endomyocardial biopsy (EMB, Section 5.6) decrease significantly after four weeks [47,49], so CMR should be used in clinically stable patients but not replace or delay EMB [50]. CMR can detect myocardial edema (intracellular and interstitial), hyperemia, increased vascular permeability or capillary leaks, myocyte necrosis, and interstitial or focal fibrosis. Edema is assessed using T2-weighted imaging, while necrosis and fibrosis are detected via late gadolinium enhancement (LGE). Expert recommendations suggest using at least two of the three Lake Louise criteria (T2 edema, T1 early gadolinium enhancement, LGE) to improve diagnostic accuracy [48].

While CMR shows high sensitivity and specificity in acute myocarditis, these values are lower in chronic inflammatory cardiomyopathy [51]. Minor inflammatory processes and viral infections, including detection of transcriptional viral activity in the myocardium, are missed by imaging alone. Therefore, in suspected virus-induced myocardial inflammation, EMB remains essential for definitive diagnosis and targeted therapy.

### 5.5. Coronary Angiography

CMR, ECG, and laboratory results cannot reliably distinguish myocarditis from myocardial infarction or ischemic cardiomyopathy. Rapid invasive testing is required to exclude these conditions before performing myocardial biopsy. Other mimicking diseases must also be ruled out.

### 5.6. Endomyocardial Biopsy

Imaging techniques (primarily echocardiography and CMR) can evaluate the extent of myocardial damage in acute and subacute myocarditis and inflammatory cardiomyopathies and largely exclude other heart diseases such as coronary artery disease, valvular defects, and storage diseases. However, for a definitive etiopathological diagnosis as the basis for causal, specific, and personalized therapy, EMB clearly remains the gold standard [48,52,53].

The reason for the necessity of biopsy diagnostics is that the pathophysiological changes in viral-inflammatory processes primarily occur at the cellular level. Their exact cause, however, can only be determined through a specific detection of the pathogen and/or inflammation in the myocardial tissue [52]. It must be emphasized that both the quality and intensity of the inflammation—including the characterization and quantification of immune cells—as well as the detection of pathogens, including the question of whether active viral replication is present, are only possible through analysis of myocardial biopsies. It is important that a sufficient number of samples are taken from as many different myocardial areas as possible to capture focal infiltrations and tissue changes. DCM-typical myocardial alterations such as fibrosis, scars, or cardiomyocyte hypertrophy can be documented more frequently in left ventricular biopsies than in right ventricular tissue samples [3,54,55]. Overall, the analysis of myocardial biopsies represents the gold standard of diagnostics in suspected acute myocarditis or chronic inflammatory cardiomyopathy as the basis for specific, causal, and personalized therapy [9,48,53].

#### 5.6.1. Histological and Immunohistochemical Diagnostics of Inflammation

**Figure 2:**
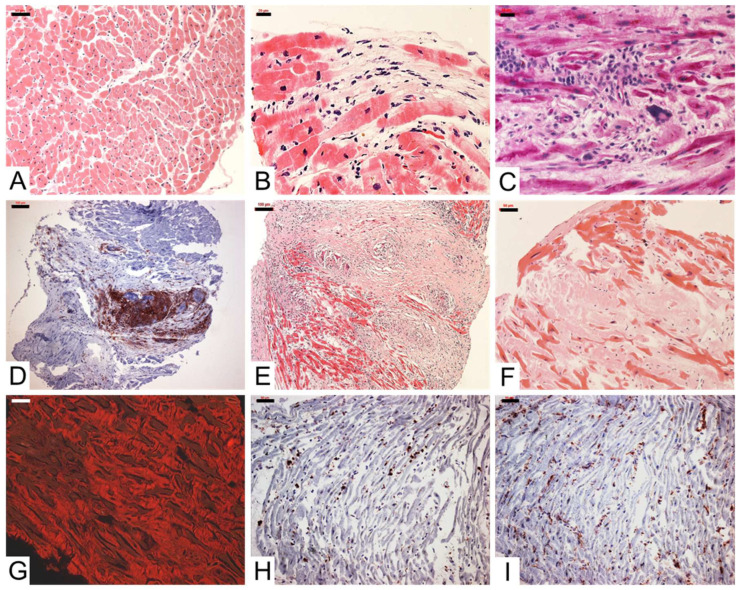
Endomyocardial biopsy inflammation diagnostics. (**A**) Hematoxylin-Eosin (H&E) staining of a normally structured endomyocardial biopsy. Myocytes arranged almost cross-sectionally in a regular pattern. (**B**) H&E staining of active myocarditis with myocytolysis. (**C**) PAS staining of active myocarditis with eosinophilic leukocytes and giant cells. (**D**) Immunohistochemical staining of CD3+ T lymphocytes in a case of borderline myocarditis with the presence of giant cells. (**E**) H&E staining of cardiac sarcoidosis with pronounced inflammatory cell infiltration, partly diffuse, partly focal. (**F**) H&E staining of cardiac amyloidosis with massive amyloid deposits in the extracellular matrix (approx. 80% of the area) and in the slightly thickened endocardium. (**G**) The same area with red fluorescence of the amyloid after Congo red staining. (**H**,**I**) Immunohistochemical staining of CD3+ T cells (**H**) and CD68+ macrophages (**I**) in inflammatory cardiomyopathy. Scale bars: (**A**) 50 µm, (**B**) 20 µm, (**C**) 20 µm, (**D**) 100 µm, (**E**) 100 µm, (**F**) 50 µm, (**G**) 50 µm, (**H**) 50 µm, (**I**) 50 µm.

Active myocarditis is present when inflammatory mononuclear cell infiltrates (lymphocytes or macrophages) are detectable together with acute myocardial cell death (necrosis), although the infiltration density of inflammatory cells is not numerically defined (Dallas criteria) (Figure 2) [56]. The new Seaport criteria from 2025 improve the histological diagnosis of lymphocytic myocarditis by incorporating standardized evaluation parameters and immunohistochemical thresholds, thereby increasing sensitivity and reproducibility compared to the traditional Dallas criteria [57]. In contrast to active myocarditis, the sole detection of inflammatory cells without myocardial cell necrosis formally corresponds to borderline myocarditis or inflammatory cardiomyopathy. If inflammatory cell infiltrates remain detectable in follow-up examinations, this is considered persistent chronic inflammatory cardiomyopathy. Since mild inflammation can only be detected to a limited extent by histological staining, sensitive immunohistochemical techniques are required. These allow for a more reliable qualitative and quantitative detection of especially chronic inflammatory processes [9]. Various immune cells are differentiated and quantified here (CD3-, CD4-, CD8-, CD45R0, LFA-1 positive lymphocytes, perforin-positive cytotoxic cells, as well as M1 and M2 macrophages) (Figure 2) [9,58,59]. Furthermore, other diagnostically and prognostically relevant parameters such as the cell adhesion molecules (CAMs) ICAM-1, VCAM-1, and HLA-DR, as well as the plasma protein plasminogen activator inhibitor type 1 (PAI-1), should be determined [9,60,61].

An increased cell infiltration of more than 7 CD3+ T lymphocytes/mm^2^ with simultaneous cellular or endothelial expression of CAMs is considered pathological in terms of a myocardial inflammatory reaction [50]. The homogeneous expression pattern of CAMs, detectable independent of the partly focal lymphocyte infiltrates, significantly contributes to the reduction in the “sampling error.” For estimating the clinical course, the characterization and quantification of different inflammatory cell subtypes is of great clinical relevance, as it closely correlates with the clinical outcome [9].

According to current guidelines, only CD3 and CD68 are recommended for detecting myocardial inflammation in EMB [48]. However, advanced immunohistochemical EMB analyses of patients with inflammatory cardiomyopathy show that other markers such as CD45R0, LFA-1, and MAC-1 also play a crucial role in the detection of inflammation [9]. For example, a significant increase in other immune cells was found in 26% of patients who underwent biopsy, even though no increase in CD3 cells was detectable [9]. Clinically and therapeutically, this means that immunosuppressive therapy should be initiated in these patients despite the absence of CD3-positive cells [62]. Without taking these markers into account, a significant number of patients with inflammatory processes would be overlooked. Several prognostic studies have demonstrated the important role of myocardial immune activation in the development of chronic heart failure [63,64,65]. Increased infiltration of CD3+ T lymphocytes in the heart consistently predicts poorer long-term outcomes, with cut-off values of approximately 13–14 cells/mm^2^ identifying patients at increased risk of progression to heart failure, cardiovascular death, or transplantation, and higher T-cell burden being associated with a significantly reduced 10-year survival rate [63,64,65]. CD45R0+ memory T cells also predict a poor prognosis [63], while the commonly used CD68+ macrophage threshold appears insufficient for risk stratification, suggesting that T cell-based quantification allows for more robust prognostic differentiation [63]. Novel studies using machine learning approaches have shown promising results regarding the prognostic role of various inflammatory markers in EMB (Section 7) [66].

Finally, special forms of acute myocarditis can be diagnosed with the help of histological analysis of myocardial biopsies. These essentially include giant cell myocarditis, eosinophilic myocarditis, and granulomatous diseases such as sarcoidosis. A major diagnostic challenge in the histological diagnosis of acute inflammatory reactions is the focal expression pattern and the associated possible “sampling error” of the biopsies, which can be overcome by gene expression profiling (Section 5.6.2).

#### 5.6.2. Analysis of Gene Expression Profiles

Through the advanced molecular biological analysis of biopsies by determining the gene expression pattern, it is possible to minimize the “sampling error” in focal processes such as in giant cell myocarditis, cardiac sarcoidosis, or eosinophilic myocarditis [29]. In a clinical study, we were able to demonstrate that the correct diagnosis of giant cell myocarditis would have been missed in 54% of cases if only histological analysis had been performed [30]. Furthermore, determining a gene expression profile can be used to monitor the success of therapy regarding both the intensity and duration of treatment. Although gene expression profiling has demonstrated high diagnostic utility in a single-center study, further multicenter studies are needed to confirm its generalizability and strengthen its external validity.

#### 5.6.3. Analysis of Cytokine Patterns in Endomyocardial Biopsies

Using an Open-Array assay, expression analyses of cytokines were performed in virus-negative patients with and without inflammation. Data analysis shows that certain cytokines—such as interleukin-1, interleukin-6, interleukin-17a, tumor necrosis factor α, and transforming growth factor β—are significantly elevated in inflammatory heart muscle diseases [67,68]. These immunological markers characterized here should serve in the future for the development of new specific therapeutic approaches, for example, through the pharmacological inhibition of cytokines using specific antibodies [69,70,71].

#### 5.6.4. Molecular Biological Virus Diagnostics

In the context of molecular biological pathogen diagnostics, nearly all relevant infectious agents (Table 1) can be detected using polymerase chain reaction (PCR) [9]. Nowadays, only a few cases of virus-associated myocarditis and dilated cardiomyopathy are caused by enterovirus infections (Coxsackieviruses A and B1-5, echoviruses), which occur with varying frequency depending on geographical location. In addition, adenoviruses (especially in children), herpes viruses, erythroparvoviruses, cytomegaloviruses (CMV), HIV, and hepatitis viruses are found in EMBs from patients with intramyocardial inflammation [9]. Multiple infections are present in approximately 30% of cases [13]. Qualitative virus diagnostics are complemented by quantitative determination of viral load using real-time PCR and sequencing of the obtained gene products for virus subtype identification. Clinical follow-up studies show that viral persistence is usually associated with progressive deterioration of left ventricular function [14,72].

Furthermore, we were able to show that the assessment of active viral replication of the currently most common virus in the myocardium—parvovirus B19 (B19V)—is of high prognostic significance, whereas the sole detection of B19V DNA in the myocardium has no prognostic relevance [72]. Evidence of an active B19V infection, which is often accompanied by inflammation, is of crucial clinical importance for treatment decisions, as immunosuppressive therapy is then contraindicated [73,74]. In contrast, initial data from clinical studies demonstrate the benefits of antiviral interferon-β therapy in the presence of an active B19V infection in adults [75] and children [76]. Conversely, immunosuppressive therapy may be administered if no active viral infection is detectable in the patient but there is pronounced intramyocardial inflammation. Several clinical studies have shown that differential therapy, depending on viral activity, significantly improve patient prognosis [13]. Recent investigations regarding intramyocardial Epstein-Barr virus (EBV) infection show similar results [77].

#### 5.6.5. Virus Diagnostics by Metagenomic Next Generation Sequencing

Since only a small number of pathogens can be examined using conventional molecular pathogen diagnostics (PCR) due to the very limited amount of available myocardial tissue, metagenomic next generation sequencing (NGS) holds great promise for improving the diagnostic utility of EMB. Advances in metagenomic sequencing enable the identification of a broader spectrum of viral genomes in clinical tissue samples, including previously unknown or unexpected pathogens [78]. Furthermore, this method can help distinguish between latent and active viral infections, which is crucial for treatment decisions [72,77]. By enriching and sequencing entire viral genome segments, it also provides insights into strain variation, classification, and potential antiviral resistance [79].

Small patient studies suggest the utility of metagenomic sequencing in the diagnosis of myocarditis and indicate potential diagnostic and therapeutic benefits [80,81,82]. The application of metagenomic NGS to serum samples from patients with myocarditis shows that viral genetic material can be detected in 41% of cases, which could not be identified using conventional diagnostic methods [82]. The viruses identified included B19V, EBV, pegivirus C, torque teno virus, and respiratory syncytial virus. In another study, Heidecker et al. performed metagenomic NGS in patients with myocarditis, analyzing peripheral blood mononuclear cells (*n* = 24), plasma (*n* = 27), EMB (*n* = 2), and explanted hearts (*n* = 13) [80]. Viral genomes identified in blood samples included B19V, EBV, CMV, pegivirus C, anellovirus, and retrovirus K, whereas tissue samples mainly contained retroviruses and only small amounts of B19V, EBV, and CMV. However, there is a lack of large-scale NGS studies using cardiac tissue that demonstrate the diagnostic utility of NGS in EMB from patients with myocarditis.

**Figure 3:**
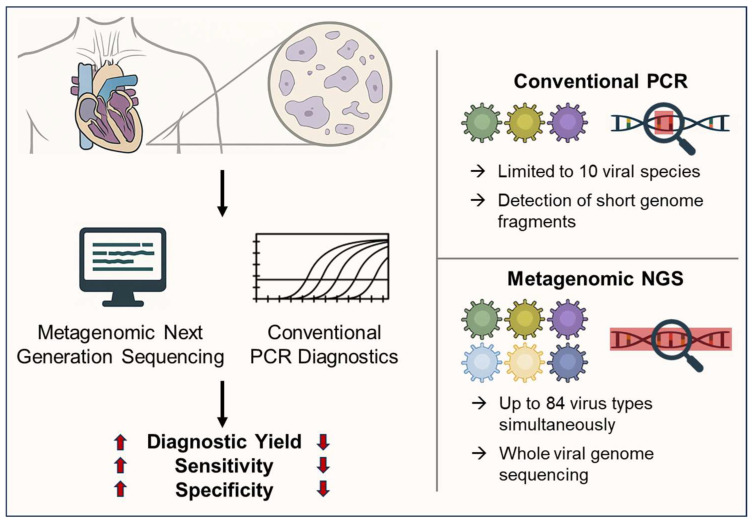
Comparison between conventional polymerase chain reaction (PCR) and metagenomic next generation sequencing (NGS) viral diagnostics. Metagenomic NGS offers superior advantages in terms of diagnostic yield (increased number of virus species) and improved sensitivity and specificity, as the entire virus genome/transcriptome is sequenced.

We have developed an NGS approach that covers 84 known and potential cardiotropic viral genomes and their transcripts and can detect them in EMB (Figure 3). Initial data show promising results regarding the detection of active viral infections [77]. However, the diagnostic and therapeutic utility of this NGS approach has yet to be demonstrated in multicenter studies.

The increased detection rate achieved through metagenomic NGS has important clinical and therapeutic implications, as patients with virus-negative myocarditis or inflammatory cardiomyopathy are typically treated with immunosuppressive therapy, whereas this approach is contraindicated in virus-positive cases, particularly when transcriptional viral activity is detected and antiviral treatment is required. Thanks to the higher sensitivity and specificity of NGS, false-negative viral findings could be minimized, new cardiotropic viruses identified, and clinically highly relevant transcriptional virus activities clearly detected (Figure 3). The NGS platform detects not only the most common cardiotropic viruses and their RNA transcripts, but also potentially new cardiotropic viruses (e.g., Zika virus, Chikungunya virus, West Nile virus, human parechovirus, dengue virus) for which routine testing is not performed. Furthermore, whole-genome sequencing has been established for the majority of pathogens, which is essential for determining genotypes and viral variants (Figure 3).

The detection of viral genomes by NGS alone does not necessarily indicate pathogenicity or justify specific therapy, especially in the absence of active viral transcription or significant myocardial inflammation. The routine clinical use of NGS is limited by several practical challenges, including higher costs, longer turnaround times, and the need for expertise and infrastructure in specialized centers with experience in molecular biology and advanced sequencing technologies. However, broader availability and lower costs are expected to facilitate future integration into routine clinical practice. Moreover, future longitudinal multicenter studies combining molecular findings with clinical course and treatment response are essential to clarify the prognostic relevance and therapeutic utility of NGS-based viral diagnostics in inflammatory heart disease.

### 5.7. The Significance of microRNAs in the Serum

MicroRNAs (miRNA) play a crucial role both in the pathophysiology and in the diagnosis of myocarditis and inflammatory cardiomyopathy [81,83,84,85,86,87,88,89]. By analyzing miRNA profiles in the serum of patients with biopsy-confirmed virus-positive or virus-negative inflammatory myocardial disease, we have succeeded for the first time in creating a serum miRNA profile that supports the suspected diagnosis of inflammatory heart disease in a non-invasive manner. This serum test can therefore be used to identify patients with inflammatory myocardial diseases. In the future, this serum assay will enable treating physicians to specifically indicate an EMB in patients with unexplained heart failure for exact characterization of the inflammation and/or viral infections. We expect that this will lead to earlier and more frequent indications for EMB, thereby significantly improving patient prognosis through early, accurate diagnosis based on biopsy analysis and enabling specific, causal, and personalized therapy [83].

### 5.8. Genetic Predisposition

It is well known that genetic predisposition appears to play a decisive role in the development of myocarditis in at least some cases. Genetic evaluation should ideally be conducted by specialists in human genetics and should include a three-generation family history [90]. Recent studies demonstrate that host genetic variation influences susceptibility, immune responses, and clinical outcomes in infectious diseases, underscoring the value of diverse population analyses, multi-omics profiling, and advanced analytical techniques for understanding pathogenesis, guiding risk assessment, and developing therapeutic strategies [91]. Furthermore, the progression of myocarditis to chronic cardiomyopathy is shaped by the complex interaction between viral infection and an individual’s genetic background, underscoring the importance of identifying genetic factors to support precision medicine and personalized treatment strategies [92,93].

## 6. Differential Diagnoses

In cases of suspected acute or chronic viral-inflammatory heart muscle diseases, various cardiac, pulmonary, and systemic conditions with similar symptomatology must be considered and, in particular, ruled out before making treatment decisions. Other forms of cardiomyopathies can also be associated with intramyocardial inflammation (Table 2). Accurate diagnosis through EMB analysis is crucial in these cases. Examples include arrhythmogenic right ventricular cardiomyopathy (ARVC), hypertrophic cardiomyopathy (HCM), and cardiac amyloidosis (Table 2) [5,7]. The underlying causes or triggers for these conditions are not yet fully understood and remain subjects of ongoing research. In the case of HCM, it is believed that disturbances of cardiomyocytes—associated with mechanical stress and sarcomere damage, mitochondrial oxidative stress, and microvascular disease with tissue injury—play a role, which carries significant prognostic relevance [94,95,96].

## 7. Artificial Intelligence as Prognostic Indicator

With the help of artificial intelligence (AI) and machine learning, it is possible to weight the parameters obtained from EMB analysis and, thus, enable early prognostic predictions [97]. This primarily involves the characterization and quantification of immune cells, as well as the detection of viruses per se and the presence of transcriptional viral activity. A recent study reported that machine learning applied to multiparametric EMB data enables accurate risk stratification in parvovirus B19-positive heart failure patients, with intramyocardial inflammation and viral activity emerging as key predictors of adverse outcomes [66]. For the first time, this allows risk stratification as a basis for precision medicine in patients with inflammatory cardiomyopathy, information that is normally not available to clinicians. This applies to the primary diagnosis, assessment of prognosis, decision-making regarding possible specific therapies, and monitoring of clinical follow-up [98,99,100,101]. It is important to note that AI-based risk stratification is a novel tool with promising potential. However, prospective multicenter validation studies are required before it can be recommended for widespread clinical use.

## 8. Therapy

### General Treatment Guidelines and Specific Therapy Regimens

**Figure 4:**
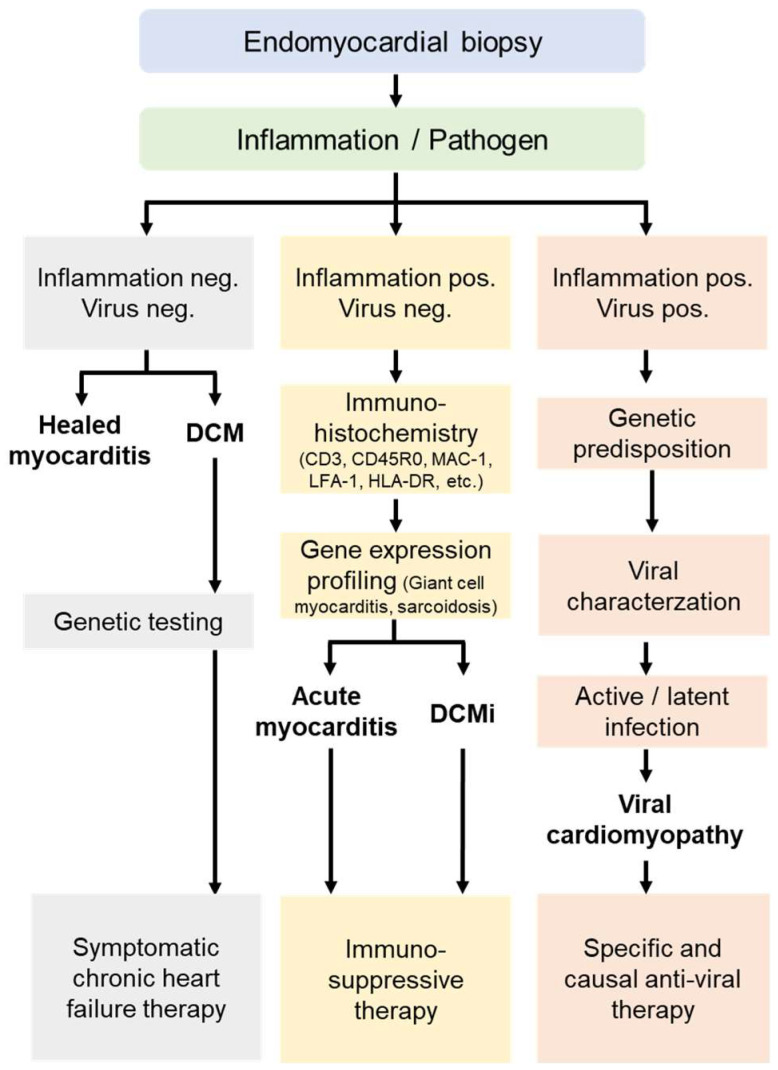
Flow chart showing the various findings of an endomyocardial biopsy examination. The detection and differentiation of the inflammatory response and the exclusion of underlying pathogens are of crucial importance. Detailed analyses such as immunohistochemical inflammation diagnostics, genetic testing, and the determination of viral activity provide information about etiology and the corresponding therapy recommendation. DCM, dilated cardiomyopathy; DCMi, inflammatory cardiomyopathy.

The treatment of heart failure or ventricular arrhythmias is always symptomatic and follows general evidence-based guidelines [102], regardless of the underlying cause. A specific treatment for myocarditis or inflammatory cardiomyopathy is only possible after determining the etiology and is based on the results of histological, immunohistological, and molecular findings from the EMB (Figure 4).

In cases of fulminant disease courses, consistent intensive care treatment and monitoring are required [48]. For fulminant myocarditis, where maximal heart failure therapies including positive inotropic agents fail to achieve stabilization, a combined therapy using IMPELLA and extracorporeal membrane oxygenation (ECMO) should be considered. Patients who cannot be stabilized and suffer from rapidly progressing heart failure that cannot be controlled either medically or interventionally must be referred to a cardiac surgery center at an early stage. Pharmacologically, in the acute phase of myocarditis, intravenous administration of immunoglobulins and high-dose methylprednisolone should be considered.

Therapy for active myocarditis or chronic inflammatory cardiomyopathy [103] should be conducted in accordance with the ESC position paper from 2013 [53] and 2025 [48], the JCS guidelines [104] and the ACC consensus paper from 2025 [1]. In active myocarditis and chronic inflammatory cardiomyopathy, immunosuppressive treatment with corticosteroids, azathioprine, or cyclosporine should only be initiated after exclusion of a viral infection. Here, it must be emphasized that viral detection is only possible through analysis of EMB.

The importance of excluding myocardial viral infection as a prerequisite for immunosuppressive therapy was demonstrated in a retrospective analysis by Frustaci et al. [73]. This study showed that patients with viral persistence who received immunosuppressive therapy experienced a significant worsening of prognosis. In contrast, the randomized, prospective, double-blind TIMIC study demonstrated that in 85 virus-negative patients with chronic inflammatory cardiomyopathy, 88% of the 43 treated patients showed a significant improvement in left ventricular function and a significant reduction in left ventricular dimensions compared to 42 placebo patients under combination therapy with prednisone and azathioprine [105]. These data confirm the effectiveness of immunosuppression in virus-negative inflammatory cardiomyopathy, which was also evident in the 20-year follow-up [62]. The 12% non-responders likely indicate the presence of an unidentified viral infection or advanced damage and inflammatory processes that no longer respond to immunosuppression.

In another non-randomized observational study involving 114 virus-negative patients with non-ischemic cardiomyopathy, these findings were confirmed. Under immunosuppressive therapy with prednisolone and azathioprine, a significant improvement in left ventricular function was observed after 6 months of treatment, increasing from 44.6 ± 17.3% to 51.8 ± 15.5% (*p* = 0.006). This improvement in LV function was also maintained in the long-term analysis for up to 10 years [106].

Recent case studies suggest that interleukin-6 and interleukin-17a inhibition could be considered as future therapeutic options for acute myocarditis and chronic inflammatory cardiomyopathy [107,108,109]. However, their benefits still need to be confirmed in randomized studies. Moreover, interleukin-1 inhibition has therapeutic potential, as high interleukin-1 levels correlate with the severity of inflammation [70,110].

Finally, it should be noted that patients with active myocarditis require close monitoring even after hospital discharge. Abstinence from sports is recommended for at least 6 months following acute myocarditis.

If viral detection is positive in the EMB, antiviral therapy should be considered. In a randomized controlled trial, we demonstrated that treatment of chronic viral cardiomyopathy with interferon-β in enterovirus and adenovirus infections resulted in clinical and hemodynamic improvements [111]. The Phase II study “Betaferon in chronic viral cardiomyopathy” (BICC) investigated the effects of immunomodulation with interferon-β therapy on viral clearance in patients with myocardial viral persistence (adenoviruses, enteroviruses, or B19V)). Study participants with enterovirus-positive or adenovirus-positive myocarditis showed viral clearance [111]. In the case of the most common virus, B19V, a retrospective sub-study of the BICC trial also demonstrated positive effects of interferon-β on left ventricular function when transcriptional viral activity was present [75].

In this context, it should be emphasized once again that the mere detection of a B19V infection in inflammatory cardiomyopathy per se has no clinical significance. Only the detection of transcriptional viral activity is associated with significant deterioration and markedly increased mortality [72]. Since intramyocardial inflammation is often observed alongside a positive B19V detection, the identification of transcriptional viral activity is of particular therapeutic importance, as immunosuppressive therapy should not be initiated in this case. In contrast, immunosuppressive therapy is possible when only latent B19V genomes are present without transcriptional viral activity. The sole detection of B19V, regardless of viral load, without transcriptional viral activity and without accompanying inflammation, has no prognostic relevance and therefore does not require specific therapy [112,113].

For other different forms of myocarditis and the specific therapy options based on the EMB results, please refer to Table 3.

## 9. Course and Prognosis

The long-term disease course of the various forms of myocarditis depends on the causative pathogen, transcriptional viral activity, the extent and quality of the inflammation, and the initial myocardial damage already present. Therefore, it is crucial to establish an early, precise diagnosis based on analyses of endomyocardial biopsies as a foundation for specific, causal, and personalized therapy.

Focal or mild inflammatory processes (affecting 60–70% of patients) often resolve spontaneously with clinical improvement of left ventricular function, especially when no significant heart failure or viral infection is present initially. However, early mortality in intensive care patients with fulminant lymphocytic myocarditis exceeds 40% within the first four weeks [118]. Untreated giant cell myocarditis or eosinophilic myocarditis show extremely poor prognoses with 4-year survival rates below 20% [119,120]. Granulomatous necrotizing myocarditis is lethal if undiagnosed and untreated.

Non-fulminant active myocarditis is burdened by progressive heart failure and sudden cardiac death, with mortality rates between 25–56% within three to ten years, especially when symptomatic heart failure is already present early on [15,24,121,122]. In the US “Myocarditis Treatment Trial” initiated in 1985, 111 enrolled patients showed a mortality rate of 20% after one year and 56% after 4.3 years, with a better prognosis emerging over longer follow-up in patients treated with immunosuppressive therapy. Besides left and right ventricular dysfunction, persistent viral infection, chronic inflammation, and cardiodepressive autoantibodies are independent predictors of poor outcome [12,123]. Biopsy-based specific treatment options are crucial to sustainably improving prognosis.

It should be noted that an initial improvement or normalization of left ventricular function during the untreated spontaneous course does not necessarily predict long-term prognosis. About 20–30% of patients, often after a relatively symptom-free and stable interval of months or years, develop life-threatening arrhythmias or progressive heart failure. This applies to both chronic viral-inflammatory cardiomyopathies and post-infectious or post-inflammatory dilated cardiomyopathies [13].

## 10. Conclusions

To conclude, a comprehensive diagnostic differentiation of pathophysiological mechanisms using histological, immunohistological, and virological/molecular biological analyses of EMB is the key to precise diagnosis, prognostic assessment, and the determination of specific, causal, and personalized therapy.

Finally, it should be emphasized that while non-invasive diagnostics, including CMR, play an important supplemental role in the early detection of myocardial inflammation, they are often insufficient to establish a definitive etiopathogenetic diagnosis. In particular, precise characterization of inflammatory quality and intensity, as well as reliable detection of viral presence and transcriptional activity, requires EMB, which remains essential for etiological clarification and for guiding specific, causal therapy.

In the future, the use of AI in the analysis of EMB will significantly improve prediction of disease course, serving as a basis for adequate, pathophysiologically justified, specific, and early therapeutic decisions.

## Figures and Tables

**Table 1:** Overview of the etiology of inflammatory myocardial diseases.

Category	Subcategory	Examples
Infectious	RNA viruses	Enteroviruses (Coxsackievirus A/B, Echovirus, Poliovirus), Dengue virus, Chikungunya virus, Rabies virus, Influenza virus A/B, Mumps virus, Human Immunodeficiency Virus (HIV), SARS-CoV-2
	DNA viruses	Adenoviruses, Erythroparvoviruses/Parvovirus B19 (B19V), Human Herpesvirus 6, Epstein-Barr Virus, Herpes Simplex Virus, Varicella-Zoster Virus, Cytomegalovirus
	Bacteria	Staphylococci, Streptococci, Pneumococci, Salmonella, Legionella, Corynebacteria, *Haemophilus influenzae*, *Mycobacterium tuberculosis*, *Mycoplasma pneumoniae*, Brucella
	Spirochetes	Borrelia, Leptospira
	Fungi	Aspergillus, Actinomyces, Candida, Cryptococcus, Histoplasma
	Protozoa	*Trypanosoma cruzi*, *Toxoplasma gondii*, Leishmania
	Parasites	Trichinella, Echinococcus
	Rickettsiae	*Coxiella burnetii* (Q fever), *Rickettsia rickettsii* (Rocky Mountain spotted fever)
Autoimmune/ Autoinflammatory		Post-infectious, Giant cell myocarditis
	Systemic autoimmune diseases	Systemic lupus erythematosus, rheumatoid arthritis, Churg-Strauss vasculitis, Wegener’s granulomatosis, sarcoidosis, myasthenia, rheumatic fever, Sjögren’s syndrome, scleroderma, polymyositis, inflammatory bowel diseases, Kawasaki syndrome
Toxic/Allergic	Medications	Penicillin, tetracyclines, cephalosporins, sulfonamides, tricyclic antidepressants, clozapine, antirheumatics, phenytoin, thiazides, furosemide, amitriptyline, lithium, lidocaine, colchicine, catecholamines, interleukin-2, trastuzumab, cyclophosphamide, cocaine, fluorouracil, ethanol, checkpoint inhibitors
	Heavy metals	Arsenic, iron, copper, lead
	Physical agents	Radiation therapy, electric shock

**Table 2:** Differential diagnoses of various cardiomyopathies.

Differential Diagnoses
Acute myocardial infarction and ischemic cardiomyopathy
Acute valvular heart disease
Toxic cardiomyopathies
Peripartum cardiomyopathy
Pericarditis
Cardiac involvement in systemic diseases
Tako-Tsubo syndrome
Tachycardiomyopathy
Non-compaction cardiomyopathy
Arrhythmogenic right ventricular cardiomyopathy (ARVC)
Hypertrophic cardiomyopathy (HCM)
Amyloidosis/storage diseases

**Table 3:** Types of myocarditis and corresponding specific therapy options [9,75,103,105,111,114,115,116,117].

Form	Definition	First-Line Therapy Option	Doses	Duration
Giant Cell Myocarditis	Myocarditis characterized by multinucleated giant cells, usually with a fulminant course and lethal if untreated.	Methylprednisolone, followed by Prednisolone with gradual dose reduction over 1 year combined with Cyclosporin A or Azathioprine or Mycophenolate Mofetil (MMF) or ATG.	1 g/day 1 mg/kg/day 2 × 75–150 mg 100 mg/day	1 year
Myocarditis/Inflammatory Cardiomyopathy (virus-negative)	Persistent/chronic myocarditis/inflammatory cardiomyopathy (>1 month symptom onset) with hypokinetic, usually dilated cardiomyopathy phenotype. Histology shows fibrosis and inflammatory infiltrates.	Prednisolone-high, then Prednisolone-low plus Azathioprine.	1 mg/kg/day 0.33 mg/kg/day 100 mg/day	4 weeks 6 months
Eosinophilic Myocarditis	Myocarditis characterized by eosinophilic infiltrates in the biopsy.	Prednisolone with subsequent dose reduction.	1 mg/kg/day −10 mg/2 weeks	9–12 months; in case of recurrence, possibly lifelong
Sarcoidosis	Cardiac involvement of systemic or isolated cardiac sarcoidosis, characterized by granulomas and chronic inflammation.	Methylprednisolone, followed by Prednisolone with dose reduction to a maintenance dose.	500–1000 mg/day 1 mg/kg/day −10 mg/4 weeks 10 mg/day	2–3 days 12–16 months; in case of recurrence, possibly lifelong
Virus-positive Myocarditis	Myocarditis with detection of viral genome in the myocardium.	For Enterovirus/Adenovirus or transcriptionally active B19V: Interferon-β-low, then Interferon-β-high. For active EBV infection possibly antiviral therapy (Acyclovir, Ganciclovir/Valganciclovir).	4 million IU *s.c.* every 2 days 8 million IU *s.c.* every 2 days	2 weeks 6 months
Immune Checkpoint Inhibitor (ICI)-induced Myocarditis	Specific form of immune-mediated myocarditis triggered by immune checkpoint inhibitors.	Methylprednisolone, followed by gradual reduction in Prednisolone and discontinuation of ICI treatment.	500–1000 mg/day	2–3 days 4–6 weeks

## Data Availability

No new data were created or analyzed in this study. Data sharing is not applicable to this article.
